# The difficult removal of tracheal tube after general anesthesia: A case report

**DOI:** 10.1097/MD.0000000000030968

**Published:** 2022-10-07

**Authors:** Man Li, YaLan Yan, PeiYu Li, Lan Zhang

**Affiliations:** a Department of Anesthesia, Sichuan Provincial Orthopedic Hospital (Chengdu Sports Hospital and Chengdu Research Institute for Sports Injury), Chengdu, China.

**Keywords:** airway management, anesthesia

## Abstract

Laryngeal injury is common after endotracheal intubation, presenting with varying degrees of edema, ulceration, granulation, and limited vocal cord movement, usually resulting in lumen narrowing. In these cases, laryngeal edema is a common complication after intubation, usually caused by direct pressure and inflammatory reaction caused by endotracheal intubation on the contact surface.

**Case presentation:**

A 71-year-old female was scheduled to undergo open reduction and internal fixation of femoral neck. On admission, she was diagnosed with femoral neck fracture. Tracheal intubation induced by general anesthesia was successful, but the tracheal catheter was difficult to remove after the operation. After 2 days of detumescence in ICU, the extubation was successful under the condition of complete recovery of spontaneous breathing.

**Conclusions:**

Patients undergoing general anesthesia may have laryngeal or glottic edema due to operation time, operation and other reasons, resulting in difficulty in extubation after general anesthesia. The extubation action shall be gentle. In case of obvious resistance, it shall not be forcibly extubated to prevent serious dyspnea after extubation.

## 1. Introduction

Laryngeal injury after endotracheal intubation is very common, manifested in varying degrees of edema, ulcer, granulation and limited vocal cord activity, usually leading to lumen stenosis. An early study published by DARMON et al in 1992 involved 700 consecutive patients who needed endotracheal intubation and mechanical ventilation. The study noted that women with ventilation duration longer than 36 hours are a risk factor for laryngeal edema after extubation.^[1,2]^ In this paper, we describe a 71 year old female patient with femoral neck fracture who underwent open reduction and internal fixation of femoral neck after anesthesia and had difficulty in removing endotracheal tube.

## 2. Case presentation

The patient was female, 71 years old, 145 cm in height and 45 kg in weight. She was admitted to the hospital because of “post-traumatic left lower limb pain accompanied by limited activity for 2 h”. After admission, she was diagnosed as “fracture of left femoral neck” and planned to undergo elective surgery. The patient denied history of heart disease and infectious diseases, and no history of diabetes, hypertension and operation and esthesia. Blood pressure (BP) was 124/69 mm Hg, heart rate (HR) was 72 times/min, pulse oxygen saturation (SpO_2_) was 96%, cardiopulmonary auscultation was not special, and no difficulty in endotracheal intubation was predicted. Chest X-ray showed a few chronic inflammatory lesions in both lungs; An electrocardiogram examination was normal. Blood biochemical determination of globulin value and ratio, creatinine, urea nitrogen were normal range. Blood routine results showed that hemoglobin was 131 g/L, hematocrit was 83%, platelet was 68 × 109/L, no thrombosis was found in deep veins of both lower limbs, American Standards Association grade ii. The left femoral neck fracture was treated by open reduction and internal fixation with endotracheal intubation combined with general anesthesia.

BP 140/80 mm Hg, HR 78 times/min and SpO_2_ 96% were measured after admission to the operating room. Electrocardiogram, noninvasive blood pressure, HR and SpO_2_ were monitored continuously. Left iliac fascial nerve block was performed with 0.2% ropivacaine 30 mL under ultrasound guidance. After 3 minutes of oxygen and nitrogen removal, sufentanil 15 µg, etomidate 10 mg, propofol 50 mg, and Rocuronium 40 mg were intravenously injected for general anesthesia induction. After the muscle relaxation was improved, id 6.5 mm reinforced tracheal catheter with steel wire was inserted through the mouth with visual laryngoscope, and the sound of both lungs was clear and symmetrical. After that, the tracheal catheter was fixed and anesthesia ventilator was used for intermittent positive pressure ventilation [Tidal Volume (VT) 320 mL, F 12 times/min]. Continuous low-flow inhalation of 1% to 2% sevoflurane and continuous micropump propofol (10–20 mL/h) during the operation maintained general anesthesia. During the operation, hemodynamics was stable and the operation was smooth. The operation lasted for 90 minutes and was completed at 19:45 on the same day. 5 minutes later, the patient’s spontaneous respiration recovered, VT was about 250 mL, neostemine 1 mg + atropine 0.5 mg antagonistic residual muscle relaxation was given, VT increased to 350 mL, the patient could raise her head, extend her tongue and clench her fist as instructed, SpO_2_ 97%, BP and HR normal after 5 minutes of spontaneous air breathing. After the tracheal and oral secretions were fully sucked out, extubation was prepared at 19:50 (140 minutes after induction and 25 minutes after operation). After the air in the balloon was drained, it was found that it was difficult to remove the tracheal tube.

Considering that the tension of vocal cords had recovered after the patient woke up or laryngeal spasm might be the cause, oxygen was given immediately and propofol 30 mg was given intravenously until the patient fell asleep and tried again, but still failed. The other two anesthesiologists failed in each attempt. At this time, it was suspected that the fine gas injection tube of the endotracheal tube cuff was blocked or indicated the failure of the one-way valve of the cuff, and the gas in the endotracheal tube cuff could not be completely pumped out. Therefore 5 mL normal saline was injected into the cuff. It was found that there was no resistance and 5 mL normal saline was successfully pumped out, thus eliminating the reason why the cuff could not be deflated.

**Figure 1.**
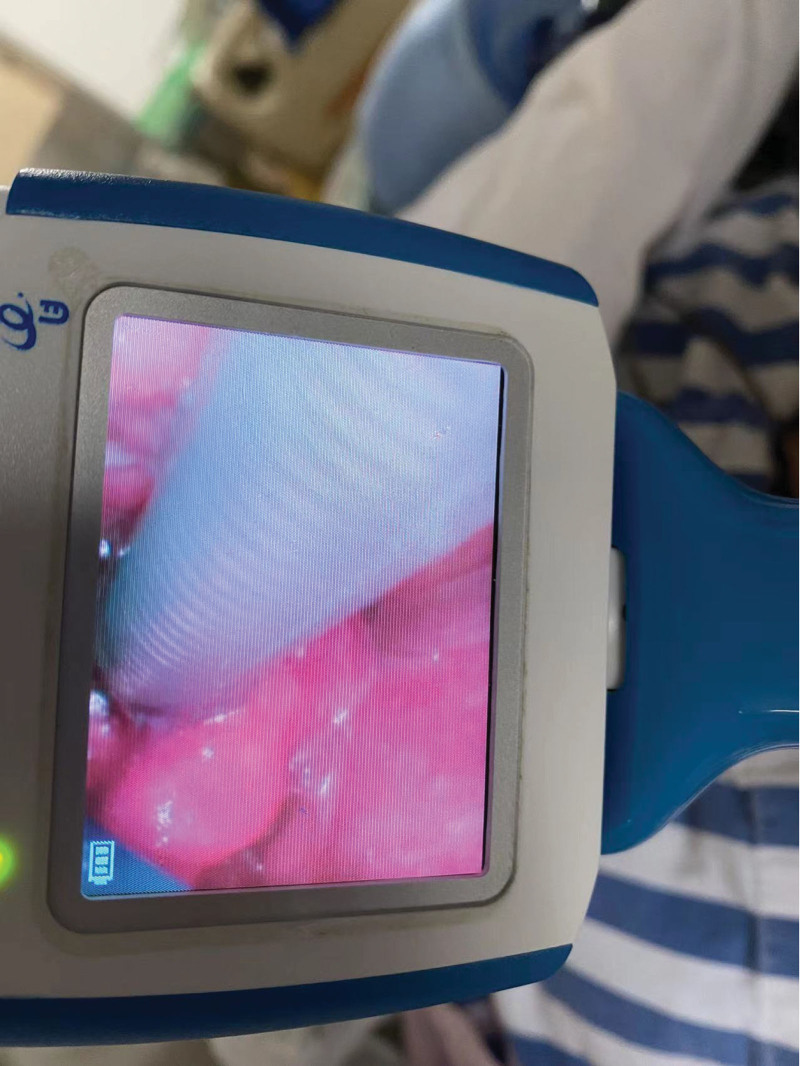
Video laryngoscopy with tube: during the tracheal tube with tube, the glottis mucosa was swollen obviously by laryngoscope.

In order to avoid vocal cord damage caused by forcible extubation, urgent request superior doctor. After the superior doctor arrived to understand the treatment process, the patient had clear breath sounds in both lungs through auscultation, and the tracheal tube was checked to be unobstructed with sputum suction tube. After another intravenous injection of 30 mg propofol and 30 mg succinylcholine, video laryngoscopy was used to examine the acoustic doorway after the patient fell asleep, and it was found that the vocal cord edema was obvious and close to the tracheal tube, and there was almost no space between the tube and the vocal cord (Fig. 1). Therefore, it was decided to take the tube temporarily to Intensive Care Unit (ICU) for observation at 20:20.

**Figure 2.**
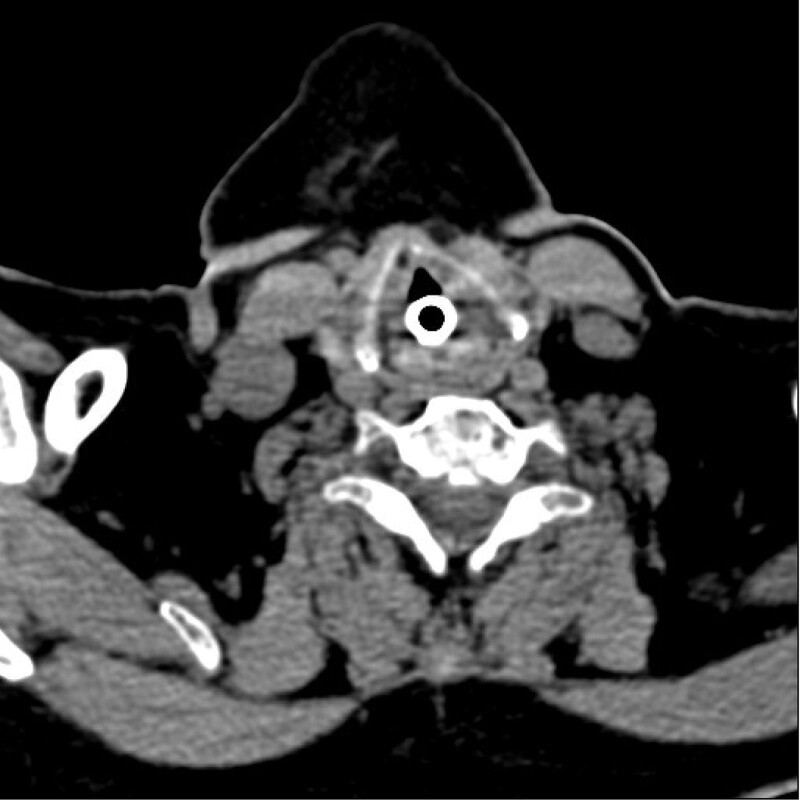
CT with tube: CT scan of the neck during tracheal tube placement showed obvious edema of the soft tissue around the glottis. CT = computerized tomography.

ICU gave patients proper rehydration, improved tissue perfusion, assisted ventilation, anti-inflammatory, reduced edema, neurotrophic, prevention, and treatment of infection, maintenance of circulation and stability of internal environment. At 8:00 the next day, after the infusion of sedative drugs was gradually reduced, the patient became conscious and resumed breathing on his own. After communication with the patient, another attempt to remove the tracheal tube failed. Ultrasound was used to scan the patient’s airway, and it was found that there was obvious soft tissue edema on both sides of the glottis, and the tracheal tube jacket was located at the position of the fifth tracheal cartilage, and no other abnormal structures were found. The computerized tomography (CT) results showed soft tissue swelling of oropharynx and larynx. Consider laryngeal, glottic and subglottic edema, and continue to use anti-inflammatory drugs to eliminate edema (Fig. 2).

The next day, the patient was awake. Under supervision, 5 mL 2% lidocaine was sprayed around the glottis of the larynx with a laryngeal anesthesia tube, and 3 mL 2% lidocaine was sprayed into the trachea through a tracheal tube. Pure oxygen was continued to be inhaled, and the glottis was observed again with a video laryngoscope 10 minutes later. At this time, a gap between the tracheal tube and glottic cleft was found, and the tracheal tube with steel wire was gently rotated under direct vision. It was found that the wrinkled and raised capsule was stuck under the right vocal cord. The catheter was continued to rotate counterclockwise so that the wrinkled and raised capsule was located above the glottic cleft. Finally, the tracheal catheter was successfully pulled out with a little resistance. Before extubation, the patient woke up perfectly and her vital signs were stable. SpO_2_ 97% when she breathed air autonomously. After catheter extraction, the patient’s voice was slightly hoarse, and there was no inspiratory wheezing. Auscultation revealed clear breath sounds in both lungs without dry and wet rale.

**Figure 3.**
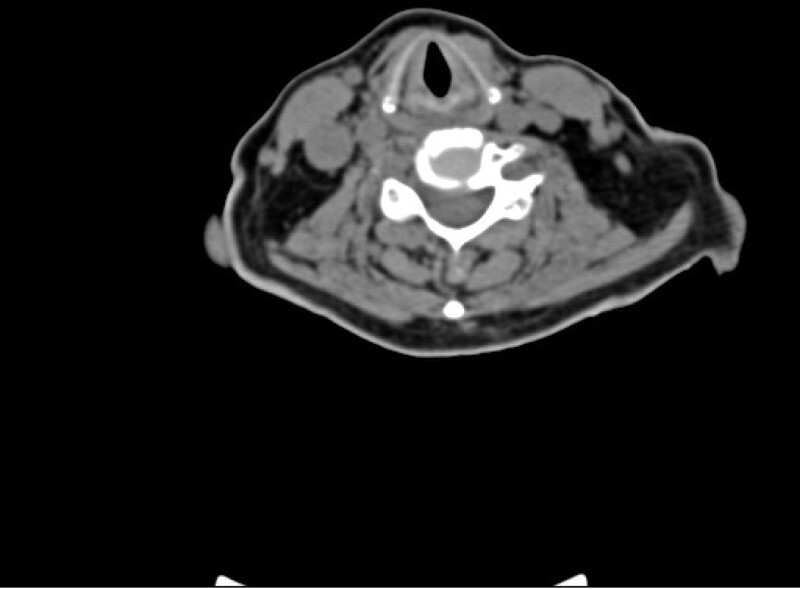
CT after extubation: CT scan of the neck after tracheal catheter extraction showed that soft tissue swelling around the glottis was reduced. CT = computerized tomography.

Three days after extubation, the neck CT showed that the soft tissue swelling near the glottis disappeared obviously (Fig. 3). The patient was discharged successfully 1 week after surgery without respiratory tract infection or complications related to endotracheal intubation.

## 3. Discussion

Tracheal tube extraction after general anesthesia is a routine operation in the department of anesthesiology. Compared with difficulty in tracheal intubation, difficulty in tracheal tube extraction is relatively rare. The most common cause of tracheal tube removal difficulty is that the catheter jacket is still inflated. The causes of severe and rare tracheal tube removal difficulty include tracheal tube fixation, sewn tracheal tube during head and neck surgery, knotted gastric tube, swelling or tense glottis, and unexpected catheter “barbs”.^[3]^ In addition, these clinical conditions are expected to be at greater risk for difficult intubation and extubation in patients undergoing head and neck surgery or prone position during neurosurgery, pregnancy, fluid resuscitation, neck and airway injuries, inhalation injuries, and burns.^[4]^ The risk of laryngeal edema after intubation can be classified into three categories: intubation factors, post-intubation factors, and patient factors. The incidence of laryngeal edema after intubation (extubation) has been reported to vary from 5% to 54%, while the incidence of wheezing after extubation has been reported to range from 1.5% to 26.3%.^[5]^

In this case, the tracheal intubation was successful, the time of surgical anesthesia was short (about 100 minutes), the process of anesthesia was smooth, and there was no allergy and other accidents. The causes of glottic edema were presumed to be improper selection of tracheal catheter (id 6.5 mm steel wire tracheal catheter was inserted in elderly women of 150 cm) and excessive tracheostomy injection and high pressure inside the tracheostomy. The glottis and tracheal mucosa tissues were compressed by high pressure in thick tracheal catheter and capsule, resulting in glottic and subportal edema. Glottic tension exacerbated the difficulty of extubation when patients woke up for extubation.

How to accurately grasp the timing of extubation is particularly important in clinical practice. Several tests have been proposed to assess airway patency before extubation. Current assessment methods include cuff leak test (CLT), ultrasound and video laryngoscopy. CLT is an important noninvasive test to assess the risk of laryngeal edema and/or wheezing after extubation in intubated patients. CLT determines the space available between the larynx and the trachea and can be quantitatively and qualitatively assessed.^[6]^ A meta-analysis of nine studies published in 2009 estimated that the sensitivity and specificity of the cuff leakage test were 56% and 92%, respectively.^[7]^ Another meta-analysis of 14 observational studies showed that although the delay in extubation increased by 9%, the cuff leakage test reduced the incidence of post extubation wheezing (4 vs 7%) and the rate of reintubation (2.4 vs 4.2%).^[8]^ Laryngeal ultrasound is a simple, rapid, and noninvasive examination that can be performed at the bedside to measure the vocal cord image Width, known as Air Column Width. This method has been proved to predict the risk of wheezing after extubation.^[9]^ A 2018 observational study of 400 children showed that the measurement of laryngeal air column width difference can be used as a simple and reliable noninvasive method to predict children’s wheezing after extubation.^[10]^ Video laryngoscope or fiberoptic bronchoscope can clearly see the structures and abnormalities around the throat and guide appropriate treatment.^[11]^

When the catheter still cannot be pulled out after cuff exhaust in clinic, violence cannot be used. The cause should be determined before other adjunctive measures including surgical extubation should be considered. Blind and excessive extubation should avoid serious complications such as vocal cord or subglottic tissue damage and tracheal bleeding.^[12,13]^

In this case, the patient had difficulty in extubation after operation, and the superior doctor decided to send the patient to ICU with a tube for anti-inflammatory and edema elimination. After comprehensive consideration, the superior doctor decided to extubation under supervision. The timing of extubation is determined for the following reasons: First, there was no problem with the tube being sent to the distal end of the patient’s tracheal catheter after the exhaust of the jacket, indicating that the patient’s lower airway was unobstructed, and the CT examination result also indicated that there was no problem with the lower airway. Second, both subglottic and distal airway leakage tests were performed, respectively. The subglottic leakage test was positive, while the distal airway leakage test was negative, proving that the patient had subglottic stenosis, but there was no problem in the distal airway. Combined with the above methods, the patient’s tracheal tube was finally safely removed.

## 4. Conclusion

There are many reasons for difficult extubation after general anesthesia. Various possible reasons should be considered and eliminated in time. Accurate operation and the implementation of anesthesia plan, gentle operation and correct and decisive clinical thinking can reduce the incidence of anesthesia related complications in patients, which is crucial to ensure the safety of patients’ lives.

## Author contributions

**Formal analysis:** Man Li.

Methodology: Man Li.

Supervision: Lan Zhang.

Writing – original draft: Man Li.

Writing – review & editing: Man Li, YaLan Yan, PeiYu Li.
